# The Use of Research Evidence in Public Health Decision Making Processes: Systematic Review

**DOI:** 10.1371/journal.pone.0021704

**Published:** 2011-07-26

**Authors:** Lois Orton, Ffion Lloyd-Williams, David Taylor-Robinson, Martin O'Flaherty, Simon Capewell

**Affiliations:** Public Health and Policy, University of Liverpool, Liverpool, Merseyside, United Kingdom; Yale University, United States of America

## Abstract

**Background:**

The use of research evidence to underpin public health policy is strongly promoted. However, its implementation has not been straightforward. The objectives of this systematic review were to synthesise empirical evidence on the use of research evidence by public health decision makers in settings with universal health care systems.

**Methods:**

To locate eligible studies, 13 bibliographic databases were screened, organisational websites were scanned, key informants were contacted and bibliographies of included studies were scrutinised. Two reviewers independently assessed studies for inclusion, extracted data and assessed methodological quality. Data were synthesised as a narrative review.

**Findings:**

18 studies were included: 15 qualitative studies, and three surveys. Their methodological quality was mixed. They were set in a range of country and decision making settings. Study participants included 1063 public health decision makers, 72 researchers, and 174 with overlapping roles. Decision making processes varied widely between settings, and were viewed differently by key players. A range of research evidence was accessed. However, there was no reliable evidence on the extent of its use. Its impact was often indirect, competing with other influences. Barriers to the use of research evidence included: decision makers' perceptions of research evidence; the gulf between researchers and decision makers; the culture of decision making; competing influences on decision making; and practical constraints. Suggested (but largely untested) ways of overcoming these barriers included: research targeted at the needs of decision makers; research clearly highlighting key messages; and capacity building. There was little evidence on the role of research evidence in decision making to reduce inequalities.

**Conclusions:**

To more effectively implement research informed public health policy, action is required by decision makers and researchers to address the barriers identified in this systematic review. There is an urgent need for evidence to support the use of research evidence to inform public health decision making to reduce inequalities.

## Introduction

In recent years, the use of research evidence to underpin public health policy has been strongly promoted. This has occurred as a natural conceptual development from the well established evidence based medicine movement [1]–[2]. In the UK, the National Institute for Health and Clinical Excellence is responsible for developing evidence based public health guidance. However, transference of the concept of “evidence based” from clinical practice to public health has not been straightforward [3], [4]. Public health decisions are taken with communities or even entire countries rather than individuals as the unit of intervention [3]. Existing evidence suggests that different parts of the population respond very differently to identical interventions [5] and an intervention that improves the health of a population may also increase inequalities in health [6]. Thus, focusing on the average effects of interventions may miss important differences [7]. Some authors argue that an evidence based approach to public health may actually increase health inequalities, as it is likely to reflect the same biases as the production of research evidence, for example favouring younger age groups, acute diseases, and drug therapy [8].

The amount and quality of research in public health is less than in clinical practice, and the certainty about effectiveness is lower [9]. Transferring the concept of “evidence based” from individuals to communities raises the importance of context and means that randomised controlled trials are frequently inappropriate [3]. Furthermore, evaluations based on prospective experimental designs are simply not possible in many areas of public health [10]. Public health decision making, and the influence of research, is also more complex. Public health policy is difficult to define as most macro policies ultimately have an effect on health [9]. Consequently, it is concerned with policy making in all fields including: fiscal, agricultural, transport, town planning, and crime [3], [11]. In the future, as methodologies for assessing the effectiveness of complex interventions are developed, the impact of such processes will become clearer.

The large number of people affected by public health policy increases the need for sound decision making. As Chalmers [12] and Macintyre and Petticrew [13] argue “good intentions and plausible theories alone are an insufficient basis for decisions about public programmes that affect the lives of others.” It has been argued that in order to develop effective public health policy, its evidence must include a wide range of influences [14]. Unlike evidence based medicine, in which randomised controlled trials and systematic reviews are mainly drawn upon, evidence for public health policy is much more complex. The policy process involves a series of steps: problem delineation, option development and then implementation. The evidence required at each step is dramatically different. Thus, public health evidence must cover, not just the question of effectiveness of interventions; but also organisation, implementation and feasibility, which are less commonly covered by research evidence [14]. In this regard, public health evidence is neither perfect, complete nor unequivocal. Research findings are so rarely definitive or robust that they rule out alternative emphases [4]. They always require interpretation in order to be implemented effectively. Suggested additional sources of evidence include: expert opinion, case study, social values and patient preferences [3], [8], [14].

Despite such complex decision making environment, until recently few primary research studies had revealed how public health decision makers used research evidence in their day-to-day work [15]. In order to synthesis newly emerging findings, we therefore decided to systematically review studies which reveal how research evidence is used by public health decision makers. There is evidence to suggest that planners and policy makers have a very different perspective when managing health care systems based mainly on private medicine, as opposed to those in which universal coverage is provided on the basis of mandatory health insurance or taxation [16]. Therefore, we explicitly limited our systematic review to countries with universal health care coverage (including: Europe, Canada, Australia and New Zealand).

### Objectives

To synthesise the evidence on how research evidence is used by public health decision makers, including:

1. the extent to which research evidence is used;
2. what types of research evidence are used;
3. the process of using research evidence;
4. factors, other than research evidence, influencing the decision making process; and
5. barriers to and facilitators of the use of research evidence.

## Methods

The review team consisted of five members, all with varied backgrounds, experiences and perspectives in public health. After developing a protocol, we undertook a comprehensive systematic review of the use of research evidence in public health decision making processes. The funders of this review, MerseyBEAT (Liverpool PCT), played no part in its design or conduct.

### Study eligibility criteria

Eligible studies must explore how research evidence is used in decision making for public health. We defined public health decision making as that which affects the general health of entire communities or populations. To be included, studies must address one or more of the five review objectives.

Studies must be based in settings with universal health care systems (including: Europe, Canada, Australia and New Zealand). Studies dating from before 1980 were excluded as these predate the establishment of the Cochrane Collaboration and the origins of evidence based medicine. No language restrictions were applied. Any study design was considered eligible, so long as it revealed empirical data relating to the review objectives.

### Search methods for identification of studies

**Figure 1:**
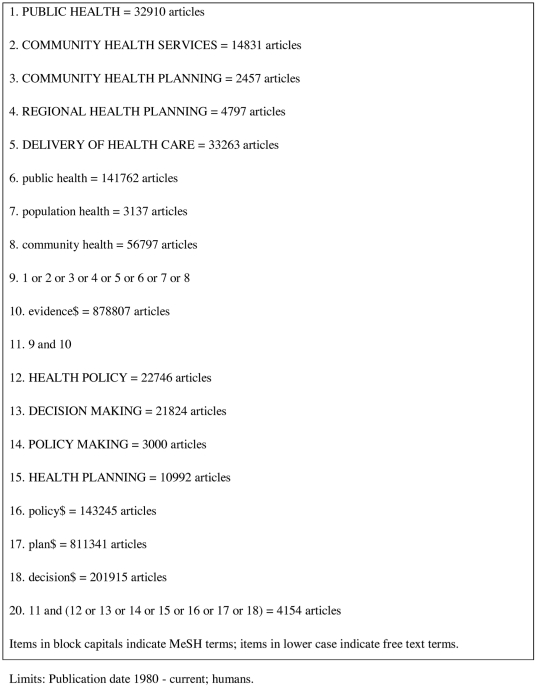
Terms used in MEDLINE search.

A search strategy was developed in order to identify relevant studies, and was adapted for each database searched (see Figure 1 for details of terms used in the MEDLINE search). Search terms were selected based on the review objectives and on the terms used to index key articles identified through early scoping searches. Databases searched from 1980 to March 2010 were: MEDLINE, SCOPUS, PsychInfo, CINAHL, The Social Science Citation Index, The Science Citation Index, The Arts and Humanities Citation Index, Applied Social Sciences Index and Abstracts (ASSIA), Database of Reviews of Effects (DARE), Cochrane Database of Systematic Reviews (CDSR), DoPHER, the Campbell Library, and the Cochrane Register of Controlled trials (CENTRAL).

General internet search engines and websites of key organisations were scanned to locate additional publications. Websites scanned were: National Health Service Knowledge, the Cochrane Collaboration, the Campbell Collaboration, the Centre for Reviews and Dissemination, Bandolier, the National Institute for Health and Clinical Excellence, the Department of Health and other public UK health related Government websites. Colleagues and key organisations working in public health policy were also contacted for any additional data sources and the reference lists of all included studies were scrutinised for other potentially eligible studies.

### Selection of studies

One reviewer screened titles and abstracts of all items retrieved to remove duplicates and to identify potentially eligible studies based on the inclusion and exclusion criteria. A sub-sample of ten per cent of these were independently screened by a second reviewer to reduce the risk of bias. All articles deemed potentially eligible were retrieved in full text. Full text articles were screened independently by two reviewers using a predesigned and piloted eligibility assessment form. Disagreements on eligibility decisions were resolved by consensus or by recourse to a third party in the review team. Details of excluded studies and reasons for their exclusion are documented in Table 1.

**Table 1:** Characteristics of excluded studies.

Reason for exclusion	Studies
Study does not relate to public health policy decision making	Abelson 2007a; Abelson 2007b; Adair 2009; Addley 1999; Aggett 2007; Allender 2009; Anderson 2006; Armstrong 2006; Armstrong 2007; Blamey 2004; Clarke 1984; Coleman 2001; de Bont 2007; Dobbins 2009; Fahey 1995; Florin 1999; Gardner 2009; Hailey 1997; Hewitt 2007; Lavis 2008a; Lavis 2008b; Millewa 2005; Mitton 2007; Morrato 2007; Nutbeam 2003; Nutbeam 2008; Renfrew 2008.
Study does not report empirical data	Asthana 2006; Davey Smith 2001; Dobbins 2002; Garvin 2001; Goodyear 2007; Graham2002; Hall 2008; Killoran 2004; Neuberger 2001; Rychetnik 2004; Stachenko 2008; Thomson 2005.
Study setting not universal health care system	Kindig 2003.

### Data extraction and management

Data from all included studies were extracted independently by two reviewers using pre-designed and piloted forms (for data extraction forms, see Text S1). Extracted data included: study design, aims, methodological quality, setting, participants, and findings in relation to the review objectives. Extracted data were compared for accuracy and completeness. Any disagreements were resolved by consensus or by recourse to a third party in the review team.

### Data synthesis

Studies included in this review were heterogeneous with diverse theoretical underpinnings. For example, in depth interview studies revealed participants' views and experiences on barriers and facilitators to the use of research evidence (objective five), and broad scale questionnaire surveys assessed the extent to which research evidence is used in practice (objective one). Data have been synthesised, and presented in the subsequent results section, separately for each review objective thus only combing data from similar studies.

Data were combined as a narrative review [17], with supporting tables. Data from individual studies were coded and organised according to the main themes identified in the systematic review objectives. Findings and interpretations are presented in the original authors' own terms without abstraction and without generating new theory. Contradictory findings are explained in terms of study design, methodological quality, and samples and settings accessed.

### Assessment of methodological quality of included studies

The methodological design of each included study, or sub-study, was categorised as either: qualitative research, quantitative research, or systematic review. Within these categories, methodological quality was assessed independently by three reviewers using tools provided by the critical appraisal skills programme [18] (Tables 2 and 3 provide details of these tools). As the included studies were diverse in theoretical underpinnings and design, and therefore not directly comparable, these tools were used to provide a qualitative assessment of study quality rather than rating the studies as high or low quality. Disagreements in methodological quality assessment were resolved by consensus or by recourse to a third party in the review team.

**Table 2:** Methodological quality of included qualitative studies.

	Behague 2008 [32]	Bickford 2008 [19]	Dobbins 2007 [20]	Elliot 200 [29]	Green 200 [33]	Harries 1999 [34]	Kapiriri 2007 [21]	Kiefer 2005 [30]	Lavis 2005 [31]	Macintyre 2001 [28]	Mitton 2004 [24]	Petticrew 2004 [26]	Ritter 2009 [25]	Taylor-Robinson 2008 [22], [23]	Whitehead 2004 [27]
**Is there a clear statement of the research aims?**	Y	Y	Y	Y	N	Y	Y	N	Y	U	N	Y	Y	Y	Y
**Is the study design appropriate?**	Y	Y	Y	Y	U	Y	Y	U	Y	U	U	Y	Y	Y	Y
**Is the recruitment strategy appropriate?**	Y	Y	Y	Y	Y	U	Y	U	Y	U	U	Y	Y	Y	Y
**Were the data collected in a way that addresses the research issue?**	Y	U	Y	Y	U	U	Y	U	Y	Y	U	Y	Y	Y	Y
**Has the relationship between researcher and participants been adequately considered?**	N	U	N	N	N	N	N	N	N	N	N	U	N	N	U
**Was the data analysis sufficiently rigorous? Is there a clear statement of the findings?**	U	Y	Y	Y	U	Y	Y	U	U	U	Y	Y	U	Y	Y
**Is there a clear statement of the findings?**	Y	Y	Y	Y	N	Y	Y	U	Y	Y	Y	Y	Y	Y	Y

Legend: Y = yes, N = No, U = Unclear.

**Table 3:** Methodological quality of included quantitative studies.

	Dobbins 2001 [38]	Dobbins 2004 [36]	Jetha 2008 [37]
**Is the study question precise?**	Y	N	U
**Is the study design appropriate?**	Y	U	U
**Is participant selection appropriate?**	Y	Y	U
**Is the exposure or intervention measured accurately?**	U	Y	N/A
**Are confounding factors taken account of in design and analysis?**	Y	Y	U
**Are outcomes measured accurately?**	Y	U	U
**Is length of follow-up adequate?**	Y	Y	N/A

Legend: Y = yes, N = No, U = Unclear, N/A = not applicable.

## Results

### The nature of included studies

**Figure 2:**
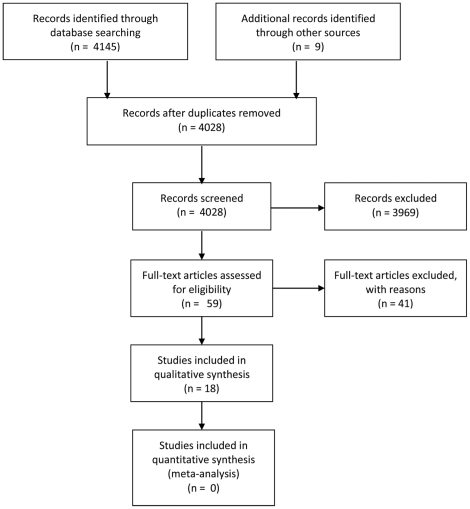
PRISMA flowchart depicting inclusion and exclusion decisions.

We identified 4154 articles from the search strategy and excluded 4095 after removing duplicates and scanning the titles and abstracts. Of the remaining 59 articles, reporting 58 studies (two articles were published from the same study), 40 did not meet our inclusion criteria (Table 1 reports the reasons for exclusion of these studies). Eighteen studies met our inclusion criteria (Tables S1 and S2 summarise their main characteristics). See Figure 2 for a flowchart depicting inclusion and exclusion decisions at each stage of assessment.

Fifteen of the 18 studies included in this systematic review had a qualitative element to their design. These included four interview studies [19]–[22]; two interview and focus group discussion studies [24]–[27]; two focussed workshops studies [26]–[27]; one study based in document analysis [28]; and six case studies using a combination of interview and review of secondary material [28]–[30], or interview, review of secondary material and observation [32]–[34]. The remaining three studies employed a quantitative survey design [35]–[37].

Of the 1309 participants in all included studies, 1063 were decision makers; 174 were involved in both research and decision making; and 72 were academic researchers. Decision makers included those at international, national, regional and local level, from public, private and third sector organisations in a range of sectors pertinent to public health (in health and beyond). Most studies were conducted in either the UK [22]–[23], [26]–[29], [34] or Canada [19]–[20], [24], [30], [36]–[38]. Three were multicentre international studies [21], [31]–[32], and one was conducted in Australia [25].

The 15 included qualitative studies addressed most, but not all, of the methodological criteria specified in the critical appraisal tool (see Table 2). No studies adequately addressed the relationship between the researcher and participants. Six [25], [28], [30]–[33] lacked sufficient information on the methods of data analysis for an assessment to be made on whether this was sufficiently rigorous. One study provided no details of interview methods or the number of participants [34]. One of the quantitative studies [37] did not provide sufficient information to make an assessment of methodological quality. The remainder addressed most of the methodological criteria for quantitative studies (see Table 3).

### The extent to which research evidence is used by public health decision makers

We found little reliable evidence quantifying the extent to which research evidence is used in public health decision making processes. A survey study published in 2001 [38] found that 63% of participating Ontario public health staff reported using at least one systematic review in the past two years to inform a decision. This study did not appear to explore the use of other types of research evidence. An Australian study also surveyed respondents to assess their use of academic research when faced with a decision making opportunity. Twenty eight per cent of public health policy makers reported using academic research [25]. However, the reliability of this finding is undermined by a lack of clarity in how data were analysed to address the research question.

### Types of research evidence used by public health decision makers

Only two qualitative studies explored the types of research evidence used by public health decision makers [20], [27]. The main findings are summarised in Table 4.

**Table 4:** Types of research evidence used by public health decision makers.

Primary research studies [20]
Systematic reviews [20]
Internal program evaluations [20]
Local and provincial best practices [20]
Observational studies that identify a problem (and in which the intervention to tackle the problem is fairly obvious) [27]
Modest, but politically timely, household studies [27]
Controlled evaluations of interventions [27]
Natural policy experiments (following the introduction of policies (in other settings) currently under consideration) [27]
Historical evidence with a long shelf life (often influences policy sub-consciously) [27]

### The process of using research evidence

Few studies revealed the process through which research evidence was used in decision making. Two qualitative studies explored how research evidence was accessed by decision makers. For Ontario provincial government workers, non-government tobacco organisations and individuals working in public health, the Ontario Tobacco Research Unit was key in disseminating research [19]. However, it is unclear if the investigators explored participants' use of other sources of research evidence. In the Australian setting, senior bureaucrats for health reported nine key sources of research evidence: experts; technical reports, monographs and bulletins (available in the unit library); the internet (particularly “Google” and clearinghouses of drug-related information); statistical data (held by the policy unit); policy makers in other jurisdictions; academic literature (used by health but not by police staff); internal expertise; government policy documents; and consultants [25].

One quantitative survey study also addressed this review objective [35]. In this study, Canadian health promotion and chronic disease prevention practitioners and policy makers consulted the following sources of evidence about chronic disease prevention and control: printed academic literature (87%); websites (85%); provincial health and recreation organisations (66%); non-government, voluntary organisations (64%); and listservs (51%). However, this study had a narrow focus (exploring the development of the Canadian Best Practices Portal) and methodological quality was unclear in most domains (see Table 3). Consequently, the wider applicability of these findings may be limited.

Five qualitative studies explored the process through which research evidence was applied in decision making. A study of Ontario public health decision makers [20] found consensus on the definition of evidence based decision making. It was generally perceived as “a process whereby multiple sources of information, including research evidence, were consulted before making a decision to plan, implement, and alter (if necessary) programs and services.” In practice, however, managers were likely to make a decision and subsequently seek evidence to justify it. Directors and medical officers saw the process in reverse, seeking evidence and then using it to inform programme decisions if applicable [20]. In Ontario and Norway the process of priority setting involved many top-down and bottom-up influences, with research evidence forming only a small part of the process [21]. For policy makers, general practitioners and researchers working on social research projects (with some responsibility for commissioning in health) research was most likely to impact on policy indirectly, shaping debate and mediating their dialogue with health service providers and users [29]. In the UK National Health Service (NHS), “organizational chaos” compounded a “labyrinthine”, rather than linear, process of change for public health [34].

### Factors, other than research, influencing public health decision making processes

Most of the included qualitative studies addressed this review objective. Interviews with UK policy makers, general practitioners and researchers with responsibility for commissioning in health revealed that research is only one of several sources of information (some of which they sought out, and some which were imposed on them) drawn upon when making decisions [30]. Other factors which influenced decisions for public health managers and policy makers in Canada and the UK included: financial sustainability, local competition, strategic fit, pressure from stakeholders, and public opinion [31]. Public health decision makers in Ontario also identified a number of sources of evidence (apart from systematic reviews and primary research studies) including: internal programme evaluations, and local and provincial best practices [22]. Policy makers in the health sector in Australia were found to review research evidence, as well as political viability, degree of community support, and other unspecified non-evidentiary aspects to decision making [25]. Health authority staff in Alberta (Canada) reported how, in the absence of good evidence, intuition, professional experience, understanding of patient preferences and other rationales such as “this has worked before…” were relied upon to make decisions. Hence, decision makers in this study suggested using a mix of “hard” and “soft” forms of evidence in priority setting [24]. Findings from this poorly reported study should, however, be interpreted with caution.

A recurring theme which emerged from a number of studies was the influence key personnel can have in the decision process, either by making judgements based on “common sense” and “expert opinion” or by acting as a filter through which evidence is transferred. Two studies explored this phenomenon in the UK NHS. They found that research evidence was only seen to affect policy with the support and commitment of those who had influence for change [34]. Rather than being a neutral tool with which to inform decision making, research evidence was in fact constructed through professional practice and contributed to the construction of professional identity [33]. The methods used in both of these studies are poorly reported. However, studies from other settings confirm the main findings. For members of Ontario tobacco control networks a large amount of tacit knowledge was held by experts in the tight knit tobacco control community. This knowledge was exchanged through dynamic, fluid and shifting networks among governmental, non-governmental and public health organisations [20]. Among Ontario public health decision makers, managers were more likely (than directors or medical officers) to connect with other colleagues to determine best practice [20]. In Australia, most senior bureaucrats in the health sector were found to consult a small group of trusted experts, some relying on this method exclusively. Experts would be contacted by phone to provide research information and opinion, resulting in quick synthesis. These experts did not need to have relevant expert knowledge, often being trusted was more important [25].

### Barriers and facilitators in the use of research evidence

The majority of included qualitative studies explored barriers and facilitators to the use of research evidence in public health decision making. Some addressed specific aspects of decision making, including: the influence of epistemology on the production and use of evidence [32]; the impact of research presentation on its use in decision making [31]; the effectiveness of current knowledge transfer processes [30]; the usefulness of models to improve decision making and priority setting [22]–[24]; and timescales for decision making [23]. Two studies specifically focussed on the production and use of research evidence to reduce health inequalities [26]–[27]. This was explored from the perspectives of international policy advisors [26] and research leaders [27].

There is a degree of consensus across studies, from various settings and including a range of different types of decision maker, on the most important factors limiting the use of research evidence in public health policy. Two studies (one with poorly reported methods) revealed a perceived lack of research evidence among public health decision makers [24], [31]. Other studies found negative perceptions of the available research evidence commonly limited its use. These included: an abundance of “policy free” evidence [26]; an undue focus on randomised controlled trials (RCTs) [32]; too much scientific uncertainty [32]; poor local applicability [24], [31], [33]; a lack of focus on the social determinants of health [26]; and a lack of complexity to address multi-component health systems [25], [32].

Three of the included studies reported a gulf between decision makers and researchers, which prevented the production of research from feeding into decision making processes [26], [32]–[33]. In two of these studies the culture within which decision makers worked lead the collection and appraisal of research to be seen as “non-work” amongst those who needed to appear to be taking action [26], [33]. Three further studies found that policy makers were not supported (through training, the structure of documents used to inform decisions, and the expectations of senior managers) to acquire the required skills or to use research evidence [24]–[25], [31].

A common finding from included studies was that competing influences, including organisational, political and strategic factors; financial and resource constraints; personal experience; common sense; expert opinion; stakeholder and public pressure; community views and local competition, restricted the use of research evidence in public health decision making [19]–[20], [24]–[25], [29], [31], [33]. Practical constraints on the use of research evidence in decision making were also commonly reported. They included: incompatible timeframes for research and policy making [19], [22]–[25], [29], [31], [34]; problems in disseminating and accessing research evidence [30]–[31]; and in its presentation (which was seen to be aimed at an academic audience) and interpretation [25], [31].

Evidence on how to overcome these barriers to the use of research evidence in public health decision making is less extensive. Included studies reported a request for improved communication and sustained dialogue between researchers and end users [27], [29], [31]–[32], [37]. In one study, the importance of trust, between researchers and policy makers was emphasized [34]. Capacity building was also seen as important to increase researchers' abilities to produce and effectively disseminate evidence of use to decision makers [30], and to improve policy makers' abilities to critically appraise and interpret these outputs [26]–[27], [31]–[32], [38]. Methodological research was thought to be needed to explore effective means of evaluating multi-component interventions [26]. In two studies it was believed that changing the culture within which policy makers work (in terms of structures, rewards and training) so that more value is placed on the use of research evidence for decisions might encourage its use [23], [38].

Some studies specified requirements for research to further inform decision making. These are outlined in Table 5. Study types which were specifically requested were varied and reflect the range of decision makers participating in the included studies. They included: “good stories”; household studies; natural policy experiments; historical evidence with a long shelf life; controlled evaluations of interventions; evidence on the costs of action or inaction; observational studies that identify a problem; predictive modelling and cost-effectiveness studies; and systematic reviews which effectively summarise evidence and increase confidence through critical appraisal [20], [26]–[27].

**Table 5:** Public health decision makers' requirements of research.

Researchers should clearly summarise their main findings [20], [25], [31].
Research approaches should show effectiveness (through study design and/or statistical presentation) and consensus [32].
Researchers should align evidence with current and future policy environments [26], [31].
Evidence must identify relevant indicators for health targets [26].
Research should make suggestions for implementation [37].
Research evidence must be designed so it is easily incorporated with colloquial/experiential/common sense knowledge [19].
Evidence is required at a local, micro level [26].
Evidence should arise from sources which are seen as unbiased (such as peer-reviewed research), authoritative and credible [19]; and provide methodological details so rigor can be assessed [37].
Funding should be provided for longer term and longitudinal research [23], [27].
Research evidence should be made more widely available to decision makers through the use of email bulletins [20], [25], public health professional organisations or clearinghouses [20].

These suggestions address some, but not all, of the barriers identified in included studies. Furthermore, their effectiveness in promoting the use of research evidence in public health decision making processes remains largely untested. This remains a research priority.

## Discussion

Results from the 18 studies included in this systematic review suggest that the process of decision making varies widely between settings, and is viewed differently by key players. An extensive range of research evidence is accessed. However, there is no reliable evidence on the extent to which it is used. Its impact is often indirect, and sits alongside many other influences. Barriers to the use of research evidence are well described and include: decision makers' perceptions of research evidence; the gulf between researchers and decision makers; the culture in which decision makers operate; competing influences on decision making; and practical constraints. Suggested (but generally untested) ways of overcoming these barriers include: research targeted at the needs of decision makers; research clearly highlighting key messages; and capacity building. There is little evidence on the role of research in influencing decision making to reduce health inequalities, a key aim of public health policy.

This systematic review outlines what is known in terms of decision making for public health in settings with universal health care systems. It goes some way to counterbalancing the North American bias in most systematic reviews of policy studies, which tend to overlook the impact of political and institutional contexts [39]. However, in order to complement the results of this systematic review, future investigators might want to synthesis studies exploring the use of research evidence in public health decision making in settings with private health care. The main strengths of the systematic review are the exhaustive search strategy, the rigorous methods used to reduce the risk of bias in the review process, and the inclusion of a wide range of qualitative and quantitative studies which reveal not only procedural aspects in the use of research evidence but also the views and experiences of various key players in the process. Despite these rigorous methods it is, however, possible that we have missed some relevant studies as much research in the social sciences is poorly indexed in bibliographic databases. Most included studies were qualitative and did not aim for representative samples. Instead, they were based in a diverse range of specific localities where public health decision making takes place. Thus, findings are not generaliseable. Clearer descriptions of participants and contexts would have helped interpret the findings from individual studies. The wide variety of study types included in the systematic review also necessitated careful consideration of methods for integrating data and for assessing methodological quality of individual studies. “Narrative review” [19] a type of “aggregative synthesis” [40]–[42] was used to summarises data, with categories being left as they were in individual included studies, rather than subsuming them at a higher level of abstraction. Aggregative syntheses have previously been criticised for being unsystematic. However, they are ideal when synthesising a wide range of different study types as their flexibility allows data from studies with a variety of theoretical underpinnings, settings, participants and outcomes to be integrated [40]. In order to enhance the reliability of this narrative review we have explicitly described the way in which the method was adopted. A wide range of tools were used to assess the methodological quality of included studies. Despite arguments for and against the usefulness and replicability of tools for qualitative studies [40], [43]–[46], most disagreements between reviewers were found to occur when methodological details were unclear rather than as a result of opposing judgements. Thus, the results of assessments appeared reliable.

The main result from this systematic review, that there are many influences (or sources of evidence) that affect public health policy decision making, reflects the findings of other published studies [11], [47] and is explained by the variety of ways in which the concept of evidence is negotiated and socially constructed by and between individuals [11], [47]. A wide range of different types of decision maker are involved in public health policy and there is the potential for endless interpretations of what evidence might constitute. Indeed, some argue that as public health policy affects a large number of people and has to be seen to be trustworthy, its evidence must include a wide range of influences such as: research evidence, expert opinion, social values and patient preferences [3], [8], [48] Tannahill [49] refers to the need for a “fuller set of measures” based on “theoretical plausibility” to complement evidence of effectiveness. Reflecting this focus, he, and others, encourage the use of the concept of “evidence informed” decision making in public health rather than the currently dominant term “evidence based.” [9], [49] Results from this systematic review, and from other studies, [50] suggest that, apart from research evidence, key personnel make an important contribution to decision making. Research evidence is considered most likely to influence policy in indirect ways, helping shape the debate along with other competing factors [30]. This fits the “enlightenment model” of the use of research evidence in decision making, which sees policy change as following a process of incremental adjustments to competing pressures, with policy evolving through an iterative process subject to continuous review [10], [51]–[55]. Klein crucially noted that “If we enlarge the meaning of evidence, there is indeed scope for bringing more intellectual edge to the analysis of what we can learn from the past [14]. But, equally important, if we remember that evidence speaks with many voices, and that our values drive facts and shape the conclusions we draw from them, we will also conclude that any such exercise will be no more, and should be no more, than one contribution to the process of policy making.”

Results from studies included in this systematic review suggest that in order to increase the use of research evidence in public health policy strategies are required to encourage two-way communication between researchers and decision makers; the environment within which decision makers work, in terms of structure and rewards, should be adapted to encourage the use of research evidence; decision makers need training to increase their ability to access and interpret research outputs; and researchers require training and support to increase their ability to produce evidence of use to policy makers, to clearly present the main findings, and to effectively disseminate them to the relevant audience. However, these suggestions do not address all of the barriers identified in this systematic review, and their effectiveness remains largely untested. Despite arguments that using research evidence might work against one of the key aims of public health policy, to reduce health inequalities [8], only two of the included studies explicitly discussed this issue. Future empirical studies testing innovations to promote the use of research evidence in public health policy should therefore take into consideration their impact on health inequalities. Furthermore, as the context of public health policy decision making varies from setting to setting, approaches to increasing the use of research evidence should follow a local needs assessment, with interventions targeted at the specific barriers identified.

In conclusion, if research informed public health is to be effectively implemented, action is urgently required by decision makers and researchers to address the barriers identified in this systematic review. There is also a pressing need for context specific evidence on the best approaches to incorporating research evidence in decision making processes that does not ignore the complex effects on health inequalities.

## Supporting Information

Table S1 **Characteristics of included qualitative studies table.**
(DOC)

Table S2 **Characteristics of included quantitative studies table.**
(DOC)
Text S1

Text S1 **Data extraction forms.**
(DOC)
