# Supplementary material for: The Use of Research Evidence in Public Health Decision Making Processes: Systematic Review
Source: PLoS One. 2011 Jul 26;6(7):e21704. doi: 10.1371/journal.pone.0021704 (PMC3144216; doi:10.1371/journal.pone.0021704)
Supplement: Table S1 — Characteristics of included qualitative studies table. (DOC) [file pone.0021704.s001.doc]

| **Study** | **Research objective** | **Setting** | **Participants** | **Methods** | **Main Findings** |
| --- | --- | --- | --- | --- | --- |
| **Behague 2008 [32]** | To identify the main epistemological barriers to evidence-based health policy-making and potential ways to overcome these. | International: maternal health. | 67 national and international researchers, United Nations agency representatives, donor body representatives, international non-governmental organisation representatives, and national-level policy experts and program managers. | Semi-structured interviews, participant observation, and review of documents. | Policy-makers held researchers responsible for paralysing action and political will by emphasising scientific uncertainty of the current evidence base. Policy-makers interested in answering health systems questions were frustrated by scientific methods’ inability to adequately address these topics. Some researchers felt that the indiscriminate use of RCTs often provided very rigorous answers to irrelevant questions. All felt that improved communication channels, more effectively disseminating new evidence, and capacity building were necessary. |
| **Bickford 2008 [19]** | To explore how research is managed, evaluated, and utilised for public health decision making. | Ontario (Canada): tobacco control. | 29 directors and policy analysts in provincial government, non-government tobacco organisation representatives with a provincial mandate, and individuals working in public health. | Secondary analysis of semi-structured interviews. | Dissonance exists between the preference for peer-reviewed, unbiased, non-partisan knowledge to support claims and the need for fast, “real time” information on which to base tobacco control policy. Difference in time-frames for research and policy decision-making is problematic. Tacit (colloquial and empirical) knowledge is held by experts and exchanged through dynamic, fluid and shifting networks. |
| **Dobbins 2007 [20]** | To identity public health decision makers’ preferences for the transfer and exchange of research knowledge. | Ontario (Canada): public health. | 16 female clinician decision makers from public health units (with a research remit) including: program managers, directors and medical officers of health. | Semi-structured telephone interviews. | Sources of evidence included: primary research studies, systematic reviews, internal program evaluations, and local and provincial best practices. Clinical expertise and past experiences important in support of decision-making. Systematic reviews were preferred as time-saving and increase confidence through critical appraisal. Request for brief research summaries and one-to-one interaction with researchers. |
| **Elliot 2000 [29]** | To identify factors that facilitate or impede evidence based public health policy making at a local level and to explore how models of research utilisation map onto empirical evidence. | UK: social research, needs and effectiveness. | 28 policy-makers, general practitioners and researchers working on case study projects, with some responsibility for commissioning healthcare (fundholders). | Nine case studies, using interview and document analysis, of social research projects initiated by NHS health authority managers and general practitioner fundholders. | Research is one of several sources of information policy makers drew on when making decisions. Factors such as financial constraints, shifting timescales and decision makers’ own experience tempered the direct influence of research evidence on decision-making. Research was more likely to impact on policy in indirect ways. Sustained dialogue between researchers and the users of research increases the use of research-based evidence in policy. |
| **Green 2000 [33]** | To explore how evidence is utilised and socially constructed in public health strategy planning. | South East of England (UK): accident prevention. | 42 members of Accident Alliances: including health authority representatives, local authority staff, health service providers, other statutory services, and voluntary agencies  2 national “policy leaders”. | Interview, observation and document analysis. | Barriers to the use of evidence included resource constraints and organisational factors. Evidence was not a neutral tool with which to inform decision-making. It was constructed through professional practice and contributed to the construction of professional identity. Collecting evidence is sometimes seen as “non-work” among “doing” professionals. Professional expertise was required to interpret evidence, and to assess the feasibility of interventions based on them. Personal experience, or “common sense”, is often valued higher than other sources of evidence. |
| **Harries 1999 [34]** | To identify factors which facilitate or impede evidence based policy making at a local level in the UK National Health Service. | UK: primary care. | Lead health authority managers, researchers, and other interested parties. | Interview, observation and document analysis. | The two worlds of research and health services management often sit uncomfortably together. In order for research evidence to be used in decision making staff in each role must share an appropriate model for research utilization. |
| **Kapiriri 2007 [21]** | To describe the process of healthcare priority setting and to identify lessons of good practice using the common conceptual framework “accountability for reasonableness”. | International (Canada and Norway). | 184 participants including: members of executive and management committees, the hospital board or administration at macro- and meso-level and health practitioners from the micro-level. | Semi-structured interview. | Differences in decision making processes were observed at the macro and meso levels. Research rarely had a major influence. |
| **Kiefer 2005 [30]** | To explore current evidence synthesis and dissemination activities for population and public health. | Canada and UK: population and public health. | 36 participants from organisations that perform systematic review or meta-analysis. | Telephone interviews and screening of organisational websites. | Population and public health researchers view knowledge exchange and uptake as a daunting challenge. Research producers lack skills, interest, or reward for knowledge exchange and uptake activities. Improved dissemination techniques, practices and evaluation should be developed considering both “producer push” and “user pull” factors. |
| **Lavis 2005 [31]** | To explore the process of health decision making, barriers and facilitators to the use of systematic review evidence in this process, and to test some innovations in the presentation of systematic reviews. | Ontario (Canada) and England and Scotland (UK). | 29 participants including: health mangers and policy-makers at government and provincial level. | Semi-structured interviews. | Many factors other than evidence influence decisions, including: financial sustainability, local competition, strategic fit, pressure from stakeholder and public opinion. The structure of documents used to inform decisions, and the expectations of senior managers, do not explicitly place a high value on research evidence. The quality of research evidence is often taken for granted, but local applicability is not. Decision makers would benefit from having information that is relevant for decisions highlighted for them. The challenge of retrieving evidence when needed was felt to remain a problem. Timelines (much tighter in policy than in research) add to this problem. |
| **Macintyre 2001 [28]** | To examine the quality of evidence underpinning the scientific advisory group to the UK’s Department of Health review of the latest available information on inequalities in health’s emerging recommendations and to identify gaps. | UK | Not applicable. | Case study involving scrutinisation, for internal and external validity, of the 17 input papers to the strategy, and their accompanying commentaries and related submissions. | There is little empirical evidence on effective interventions to reduce inequalities. There is no data on harms, costs or priorities. Upstream (whole community) interventions are least backed-up by evidence of effectiveness (when compared to downstream, Individual-level, interventions). |
| **Mitton 2004 [24]** | To explore what decision makers think is important in the application of evidence to support public health priority setting before and after the implementation of a priority setting model. | Alberta (Canada). | 8 senior managers and clinicians working at a health authority took part in two focus groups, and 17 took part in interviews. | Participatory action research including: reflection through focus groups, interviews, development and implementation of a priority setting model, and framework refinement. | Most decision makers were motivated to use evidence to inform service delivery and priority-setting. However, in the absence of good evidence, they felt that intuition, professional experience, understanding of patient preferences and other rationales such as “this has worked before...” could be relied upon. Barriers to the use of evidence included: the culture of policy-making, the crisis-oriented nature of management; time constraints, difficulty in applying evidence in the local setting, and a lack of supportive structures or skills. Decision-makers felt a lack of evidence for macro-level decisions (on broader determinants of health). |
| **Petticrew 2004 [26]** | To explore with UK and international policy advisors how research evidence influences public health policy making, and how its relevance and utility could be improved. | International (UK and Europe): health inequalities. | Seven senior policy advisors using public health research evidence and interpreting it for ministers and other senior civil policy advisors. | Four two-day workshop sessions. | Research evidence was sometimes used to justify policy decisions already taken. Evidence at a local level was requested. A number of problems with the “policy-free” nature of evidence were highlighted. The timing of evidence presentation was important. “Good stories” were particularly valuable, as was evidence on the costs of action or inaction. More evidence was requested on the social determinants of health and how to reduce health inequalities. Predictive research (such as modelling) was also requested. Methodological research was required to develop methods of evaluating “clusters of interventions”. Help was needed to deal with the “mixed economy” of evidence. |
| **Ritter 2009 [25]** | To better understand how public health policy-makers access research evidence when faced with a decision making opportunity. | Australia: health and police. | 31 senior bureaucrats. | Individual and group interview. | Academic literature was consulted in 28% of cases. Decision makers want to find ways of using research evidence that does not ignore the complexity, which must be authoritative, credible, and thorough. Apart from research, political viability, degree of community support, and other non-evidentiary aspects to decision-making are also important. When more time was available policy makers engaged in a comprehensive approach to reviewing and synthesising the research evidence. However, under the usual rapid system, their ability to do this was limited. They need easily accessible evidence sources. Nine key sources of research evidence were identified. Most also consult experts. Respondents preferred summative information. |
| **Taylor-Robinson 2008 [22, 23]** | To explore attitudes to the use of models for coronary heart disease to support decision making for policy and service planning, and to explore issues relating to timescales within which public health decisions are being made. | UK (England, Scotland and Wales): national health service, cardiovascular disease, public health. | 33 participants including: national, regional and local level decision makers; physicians; academics and voluntary organisations. | Semi-structured interviews. | Reported timescales for decision-making varied, but were typically short at around 1 to 3 years. Timescales were thought to be too short as a result of the way the organisational “system” was set up, and due to difficulties in long term forecasting. Models were not used routinely by decision makers. Those at national and regional levels were more likely to use them than those at local level. Participants felt that, in the absence of sufficient evidence to support policy-making, models could make decision making processes more evidence-led. Advantages and disadvantages of using models to support decision making are outlined. |
| **Whitehead 2004 [27]** | To explore research leaders’ perceptions and experiences of the types of evidence that influence policy on health inequalities, and their reflections on how the flow of such research evidence could be increased. | International (UK and other). | Nine senior research leaders with over 10 years’ experience of synthesising and evaluating the evidence on major policies related to health inequalities, and being current leaders in their field. | Four two-day workshop sessions. | Lists types of evidence identified as having an impact on policy. To make decision making more research informed better summation and dissemination of findings was required as was the ability of researchers and policy-makers to become more closely engaged spending a period of time working in each others’ fields and consulting at the earliest possible phase of a research tender. |
