# Supplementary material for: The Use of Research Evidence in Public Health Decision Making Processes: Systematic Review
Source: PLoS One. 2011 Jul 26;6(7):e21704. doi: 10.1371/journal.pone.0021704 (PMC3144216; doi:10.1371/journal.pone.0021704)
Supplement: Table S2 — Characteristics of included quantitative studies table. (DOC) Text S1 [file pone.0021704.s002.doc]

| **Study** | **Research Objective** | **Setting** | **Participants** | **Methods** | **Main findings** |
| --- | --- | --- | --- | --- | --- |
| **Dobbins 2001 [38]** | To determine: the extent to which systematic reviews of public health interventions influence public health decisions, and factors associated with influencing these decisions. In order to assess this, five relevant systematic reviews were disseminated to public health decision makers two years before the study. | Ontario (Canada): public health. | 141 medical and associate medical officers of health, program directors and program managers responsible for making public health decisions. | Cross-sectional telephone survey and self-administered organisational demographic questionnaire. | 89/141 (63%) participants reported using at least one systematic review in the past two years to make a decision. The percentage of retrieved articles read in a month, the value organisations placed on using research evidence for decision-making, mechanisms that facilitated the transfer of new information into the public health unit and making collaborations with other community organisations were the most significant predictors of the influence of systematic reviews on decision making processes. |
| **Dobbins 2004 [36]** | To determined whether the results of recently completed systematic reviews were used in the development of new provincial policies for public health practice. | Ontario (Canada): public health. | 51 members of technical review groups who were updating guidelines including: program managers and directors, epidemiologists, medical officers, provincial consultants, and local board of health members. | Telephone administered cross-sectional questionnaire survey. | 82% reported having used systematic reviews in the past. 59.2% indicated that they were very important compared to other forms of evidence; 2% thought their importance was negligible. The majority (61%) saw the executive summary as the most important component. 96.1% reported that results of systematic reviews played a part in the development of the new public health guidelines; data were missing for the remainder. 46.9% reported that the results completely or very extensively led to new recommendations for practice; and 2% reported that they were not used at all. |
| **Jetha 2008 [37]** | To provide an overview of the development of the evidence-based Canadian Best Practices Portal for public health. | Canada: health promotion and chronic disease prevention. | 498 health promotion and chronic disease prevention practitioners and policy-makers from all provinces of Canada including: local/regional public health departments or health authorities staff, federal/provincial/territorial government staff, non-governmental organisation staff, university employees, hospitals staff and private sectors workers. | Online survey. | Sources of evidence consulted by decision makers included: printed academic literature (87%); websites (85%); provincial health and recreation organisations (66%); non-government, voluntary organisations (64%); listservs (51%); and other sources (16%). |
