# Supplementary material for: The Use of Research Evidence in Public Health Decision Making Processes: Systematic Review
Source: PLoS One. 2011 Jul 26;6(7):e21704. doi: 10.1371/journal.pone.0021704 (PMC3144216; doi:10.1371/journal.pone.0021704)
Supplement: Text S1 — Data extraction forms. (DOC) [file pone.0021704.s003.doc]

**DATA EXTRACTION FORM**

Intervention studies

| Study ID |  | Extractor ID |  |
| --- | --- | --- | --- |
| Main author |  | Date |  |

Study aim

| Describe study aim |
| --- |
|  |

Methods

| Describe study design |
| --- |
|  |

Participants

| Participant inclusion criteria | Participant characteristics and setting |
| --- | --- |
|  |  |

Were all treatment groups comparable at baseline? **Yes No Unclear**

If **No** or **Unclear**, please describe any differences:

Notes:

Methodological quality

# Randomisation

Study randomised? **Yes No Unclear**

Generation of allocation sequence: **Adequate Inadequate Unclear**

Concealment of allocation sequence: **Adequate Inadequate Unclear**

Randomisation method:

Allocation concealment method:

# Blinding

Participant blinded? **Yes No Unclear**

Treatment provider blinded? **Yes No Unclear**

Outcome assessor blinded? **Yes No Unclear**

Data analyst blinded? **Yes No Unclear**

# Participant attrition

Number assessed for eligibility:

|  | Group I | Group II | Group III | Group IV |
| --- | --- | --- | --- | --- |
| Randomised or allocated |  |  |  |  |
| Discontinued intervention |  |  |  |  |
| Analysed |  |  |  |  |

Intention-to-treat analysis: **Yes No Unclear**

Overall follow-up status: **Adequate (>90%) Inadequate (<90%) Unclear**

Notes:

Interventions

| Group I | Group II | Group III | Group IV |
| --- | --- | --- | --- |
|  |  |  |  |

Notes:

Outcomes

| Outcome | Group I | Group II | Group III | Group IV | Significance tests |
| --- | --- | --- | --- | --- | --- |
|  |  |  |  |  |  |

Notes:

**DATA EXTRACTION FORM**

Survey studies

| Study ID |  | Extractor ID |  |
| --- | --- | --- | --- |
| Main author |  | Date |  |

Study aim

| Describe study aim |
| --- |
|  |

Methods

| Describe study design and setting |
| --- |
|  |

Participants

| Participant characteristics |
| --- |
|  |

Methodological quality

[Questions to be answered in conjunction with the CASP tools for case control studies and cohort studies]

Is the study question precise? **Yes No Unclear**

Is the study design appropriate? **Yes No Unclear**

Is participant selection appropriate? **Yes No Unclear**

Is the exposure or intervention measured accurately? **Yes No Unclear**

Are confounding factors taken account of in design and analysis? **Yes No Unclear**

Are outcomes measured accurately? **Yes No Unclear**

Is length of follow-up adequate? **Yes No Unclear**

Notes:

Results

| Results |
| --- |
|  |

Notes:

**DATA EXTRACTION FORM**

Qualitative studies

| Study ID |  | Extractor ID |  |
| --- | --- | --- | --- |
| Main author |  | Date |  |

Study aim

| Describe study aim |
| --- |
|  |

Methods

| Describe study design |
| --- |
|  |

Participants

| Participant characteristics |
| --- |
|  |

Notes:

[Questions to be answered in conjunction with the CASP tools for qualitative studies]

Is there a clear statement of the research aims? **Yes No Unclear**

Is the study design appropriate? **Yes No Unclear**

Is the recruitment strategy appropriate? **Yes No Unclear**

Were the data collected in a way that addresses the research issue?

**Yes No Unclear**

Has relationship between researcher and participants been adequately considered?

**Yes No Unclear**

Was the data analysis sufficiently rigorous? **Yes No Unclear**

Is there a clear statement of the findings? **Yes No Unclear**

Notes:

Findings

| Describe the main findings |
| --- |
|  |

Notes:

**DATA EXTRACTION FORM**

Systematic reviews

| Study ID |  | Extractor ID |  |
| --- | --- | --- | --- |
| Main author |  | Date |  |

Review aim

| Describe review aim |
| --- |
|  |

Methods

| Describe review inclusion/exclusion criteria |
| --- |
|  |

| Describe search strategy |
| --- |
|  |

| Describe screening and data extraction procedures |
| --- |
|  |

| Describe methods of data synthesis |
| --- |
|  |

Methodological quality

[Questions to be answered in conjunction with the CASP tools for systematic review]

Is there a clear statement of the review aims? **Yes No Unclear**

Did the reviewers include the right type of study? **Yes No Unclear**

Is the search strategy appropriate? **Yes No Unclear**

Did the reviewers make attempts to reduce bias in the review process (such as screening and extracting data in duplicate)? **Yes No Unclear**

Were the included studies assessed for quality? **Yes No Unclear**

If the data were synthesised, was this appropriate? **Yes No Unclear**

Notes:

Findings

| Describe the main findings |
| --- |
|  |

Notes:

**DATA EXTRACTION FORM**

Survey studies

| Study ID |  | Extractor ID |  |
| --- | --- | --- | --- |
| Main author |  | Date |  |

Study aim

| Describe study aim |
| --- |
|  |

Methods

| Describe study design and setting |
| --- |
|  |

Participants

| Participant characteristics |
| --- |
|  |

Notes:

Methodological quality

Is the study question precise? **Yes No Unclear**

Is the study design appropriate? **Yes No Unclear**

Is participant selection appropriate? **Yes No Unclear**

Is the exposure or intervention measured accurately? **Yes No Unclear**

Are confounding factors taken account of in design and analysis?

**Yes No Unclear**

Are outcomes measured accurately? **Yes No Unclear**

Is length of follow-up adequate? **Yes No Unclear**

Notes:

Results

| Results |
| --- |
|  |

Notes:
